# Associations between challenging parenting behavior and creative tendencies of children: the chain mediating roles of positive emotion and creative self-efficacy

**DOI:** 10.3389/fpsyg.2024.1255773

**Published:** 2024-04-12

**Authors:** Dasheng Shi, Yidi Wang, Ruining Jin, Lin Chu

**Affiliations:** ^1^School of Education, Minzu University of China, Beijing, China; ^2^School of Civil, Commercial and Economic Law, China University of Political Science and Law, Beijing, China; ^3^Teachers’ College of Beijing Union University, Beijing, China

**Keywords:** challenging parenting behaviors, creative tendencies, positive emotions, self-efficacy, SEM

## Abstract

**Background:**

Parenting behavior has been reported to be closely associated with children’s creativity, yet the association between challenging parenting behavior and children’s creative tendencies, as well as the potential mechanisms connecting the two, remains ambiguous. Based on the Social Cognitive Theory and the Self-efficacy Theory, this study aims to examine the correlation between Chinese parents’ challenging parenting behaviors and their children’s creative tendencies, as well as the chain mediating role of children’s positive emotions and creative self-efficacy.

**Methods:**

In total, 2,647 families were surveyed with questionnaires completed by parents on the Challenging Parenting Behaviors Scale and by children on the Positive/Negative Emotions Scale, the Creative Self-efficacy Scale, and the Williams Creative Tendency Test Scale, and analyzed using structural equation modeling (SEM) in SPSS 22.0 and Mplus 8.3.

**Results:**

The findings indicate that challenging parenting behavior has a positive correlation with children’s positive emotions, creative self-efficacy, and creative tendencies. Through positive emotions, creative self-efficacy, and a chain mediated pathway between these two variables, challenging parenting behaviors increase children’s creative tendencies.

**Conclusion:**

The favorable impacts of challenging parenting behaviors on children’s creative tendencies, with the mediating effects of children’s positive emotions and creative self-efficacy, may help Chinese parents better grasp the mechanisms underlying this association.

## 1 Introduction

Over the years, traditional beliefs such as “shun tian cong ren” (obedience to authority) and “ting fu mu yan” (listening to parents’ words), traditional educational concepts such as emphasis on discipline, conformity, and mastery of knowledge and experience, as well as an education assessment system centered around standardized exams, have to some extent led to the neglect of fostering children’s creativity and independent thinking in Chinese education. Creativity is one of the fundamental competencies of the 21st century. Childhood is a critical time for developing and fostering creativity (Alfonso-Benlliure et al., 2013), but the ease and naturalness of creativity development in childhood have been generally overestimated (Albert, 2010). Studies have shown that stimulating children’s imagination and creativity can help them develop their interests and talents, improve their critical thinking and problem-solving skills, and lay the groundwork for their future individual development and social adaptation. Therefore, it is of great significance to grasp this critical period to improve the current situation of creativity development of Chinese children. More focus should be placed on children’s “creative tendencies” than on their “creative thinking” and “creative achievement” (Runco, 2003). The familial environment of childhood has a significant impact on the creative development of children. Parents are the primary nurturers and participants in a child’s development, and children are invariably influenced by their parenting concepts, styles, and specific behaviors (Ngaosusit, 2005; Martin et al., 2007). Numerous studies have demonstrated that children’s creative tendencies can be fostered through education (Dong, 1985). Specifically, the influence of parenting behaviors in the family on children’s creative tendencies is essential for enhancing children’s willingness to innovate and improving their creative abilities. Therefore, it is crucial to investigate the mechanisms that foster children’s creative tendencies in the family setting.

The significance of the early familial environment for children’s creative potential has been well-established (Sternberg and Lubart, 1996; Jankowska and Karwowski, 2018). As an essential factor in the early family environment, parenting style is closely related to the creative development of children (Gralewski and Jankowska, 2020). With the acceleration of China’s modernization process, changes in the family structure, and the renewal of values, the roles and interactions in Chinese families have changed, and the association between Chinese parents and their children has become more equal and looser, and the parenting concepts of Chinese parents have gradually shifted from being strict and authoritative to being understanding, respectful and supportive. Especially in the context of China’s rapid economic and social development, the creative ability, which has an important impact on children’s future development, has been increasingly emphasized by Chinese parents. As a result, the new generation of Chinese parents tend to choose positive parenting styles that are more open and respectful of children’s independence and individuality. According to existing studies (Grossmann et al., 2010; Fletcher et al., 2013; Fliek et al., 2015), challenging parenting behavior is a relatively new type of parenting behavior, particularly parenting behavior that encourages and stimulates children to engage in risk-taking behaviors within the safe range by encouraging, scaring, teasing, chasing, and beating children in games, which encourages children to continuously push their limits and step outside of their comfort zone (Bögels and Phares, 2008), thus cultivating children’s bravery, confidence, risk-taking spirit, initiative, and exploration. Short videos of young Chinese parents “tricking” their children are common on the Internet, reflecting the fact that this kind of parenting behavior is becoming more and more common among the new generation of Chinese parents. Meanwhile, the correlations between positive parenting styles, creative tendencies, positive emotions, and creative self-efficacy have each been well researched in previous studies (Lim and Smith, 2008; Miller et al., 2012; Mehrinejad et al., 2015; Lazarus et al., 2016; Gralewski and Jankowska, 2020; Dechaume and Lubart, 2021). However, there is a dearth of research that examines challenging parenting behaviors and children’s creative tendencies, as well as the mediating roles of positive emotions and creative self-efficacy, from an integrated perspective of the external environment and internal psychological motivation. According to the study, some parents give their children a great deal of freedom to explore new things, whereas others restrict their children with strict rules (Maccoby and Martin, 1983). This suggests that parenting behavior is a significant external environmental factor that influences children’s creative tendencies. Challenging parenting behaviors, as positive parenting behaviors, have a positive effect on children’s creative tendencies. Positive emotions and creative self-efficacy are internal psychological factors that influence creative tendencies. The mechanisms by which external environmental factors and internal psychological factors together influence children’s creative tendencies are unproven. Notably, the majority of previous studies have only examined the inhibiting effects of challenging parenting behaviors on children’s negative emotions but have neglected to examine their potential impact on children’s creative tendencies.

Based on social cognitive theory and self-efficacy theory, this study aims to examine the association between challenging parenting behaviors and children’s creativity tendencies in the context of Chinese parents’ parenting concepts, which have evolved to focus more on positive parent–child communication, interaction, and respect due to social development and cultural changes. This will contribute to a deeper understanding of the longitudinal impact of parental caregiving on children’s development and provide practical recommendations for scientifically nurturing parents. Furthermore, to uncover the underlying mechanisms of fostering early childhood creativity tendencies, this study aims to explore the mediating factors (i.e., positive emotions and creative self-efficacy) between challenging parenting behaviors and children’s creativity tendencies. This will provide valuable insights at the family level to guide parents in China and other countries to promote children’s motivation for innovation and the development of their creative abilities.

### 1.1 Challenging parenting behavior and creative tendencies

Parenting behavior is one of the most influential factors in the creative development of children (Yeh, 2004). Parenting behavior, as a concrete expression and practice of parenting style, is essentially a daily interaction between parents and their children (Jankowska and Karwowski, 2018), in which parents repeatedly transmit their approval of their children and their own perceptions to children, thus promoting or inhibiting the skills and traits of children’s creativity (Kwaśniewska et al., 2017). According to Majdandžić et al. (2016), challenging parenting behavior is a positive parenting behavior that includes both physical play (e.g., playful games, competitive games, etc.) and social-emotional aspects (e.g., social bravery, encouragement of assertiveness and performance, etc.). It has been demonstrated that attitudes and behaviors in parent–child interactions are significant predictors of children’s creative abilities (Csikszentmihalyi, 1997; Gute et al., 2008; Kwaśniewska et al., 2017). This is because parents with challenging parenting behaviors typically provide their children with a relaxed, unrestricted, and vivacious home environment, which contributes to the development of creative activities and encourages creativity in children (Heinert, 1983). As a form of affective creativity, creative tendency consists of the four characteristics of risk-taking, curiosity, imagination, and challenge (Williams, 1980), is similarly influenced by the external environment. According to the Social Cognitive Theory (Bandura, 1997), the intrinsic motivations that influence an individual’s behaviors are supported or hindered by the external environment. When the external parenting environment is authoritarian and oppressive, children lack self-confidence, struggle to exercise autonomy, and have a suppressed propensity to innovate (Fearon et al., 2013). In contrast, children are more likely to think and act creatively when the external parenting environment is free and pleasurable (Hein et al., 2014). Therefore, this study proposes research hypothesis 1: challenging parenting behaviors positively contribute to children’s creative tendencies.

### 1.2 Challenging parenting behavior, positive emotion, and creative tendencies

Children’s positive emotions may act as a mediator between challenging parenting behaviors and children’s creative tendencies. According to the Social Cognitive Theory, parenting behaviors are goal-directed and non-goal-directed behaviors demonstrated by parents in specific contexts during parent–child interactions that influence children’s cognition, emotions, and behaviors directly (Berkien et al., 2012). It has been shown that parenting styles in the parenting environment are closely related to the development of emotional behavior in children (Eisenberg et al., 1998), specifically, parental attention, encouragement, and positive support can stimulate positive emotions in children, whereas excessive restriction, control, and punishment can inhibit positive emotions in children or even cause emotional problems. Similarly, it has been demonstrated that challenging parenting behaviors are effective in preventing the development of negative emotions in children. For instance, both Smout et al. (2020) and Möller et al. (2016) concluded that challenging parenting behaviors predicted children’s anxiety levels and that children exhibited reduced anxiety levels when their parents, particularly their fathers, adopted challenging parenting behaviors. Additionally, parenting styles and behaviors produce a corresponding emotional climate. According to the definition of challenging parenting behaviors provided by existing studies, challenging parenting behaviors are clearly positive parenting behaviors that emphasize support, encouragement, and recognition (Majdandžić et al., 2016). Therefore, parents with challenging parenting behaviors can form positive emotional interactions with their children by expressing and transmitting positive emotional messages to them during parent–child interactions, thus allowing children to gain confidence and courage from them, stimulating their curiosity and imagination, and bringing them positive emotion. Studies have confirmed that the experience of frequent positive emotions serves to broaden humans’ thoughts and behaviors (Isgett and Fredrickson, 2004), and positive emotions influence an individual’s ability to focus on the task, ability to learn, and confidence (Langebćk et al., 2012), thus creating conditions for individuals to think and act creatively. In summary, this study proposes research hypothesis 2: children’s positive emotions mediate the association between challenging parenting behaviors and children’s creative tendencies.

### 1.3 Challenging parenting behavior, creative self-efficacy, and creative tendencies

Creative self-efficacy may serve as a crucial mediator between challenging parenting behaviors and children’s creative tendencies. Bandura pioneered the concept of self-efficacy in 1977; subsequently, Tierney and Farmer (2002, 2004) further clarified the concept of creative self-efficacy, defining it as the individual’s beliefs and expectations regarding their own creative performance in creative activities. The influence of intrinsic motivation on the propensity to be creative is significant (Runco and Nemiro, 1994). As one of the most important internal motivators of individuals, creative self-efficacy is an intrinsic drive that propels and sustains individuals in creative activities, and it determines the degree of psychological tendency and behavioral effort to engage in creative activities. Numerous studies have shown that people with high creative self-efficacy are usually highly creative (Choi, 2004; Gong et al., 2009) and creative self-beliefs influence individuals’ decisions to make creative moves (Dollinger, 2011), this means that people who believe they have creative potential will put more effort into creative tasks (Schack, 1989; Choi, 2004), and are characterized by creative engagement in their work (Carmeli and Schaubroeck, 2007; Tierney and Farmer, 2011). It is evident that self-perceptions and confidence in one’s abilities influence an individual’s thoughts, emotions, and behavioral tendencies. Children with low creative self-efficacy typically lack self-confidence, choose to escape when they encounter frustration, become more skeptical of their ability to generate creative ideas, and are consequently reluctant to actively seek effective solutions to problems, thereby stifling their creative tendencies and reducing their own creativity. In contrast, children with high creative self-efficacy have more positive self-evaluations and participate in a wider range of activities (Atwood-Blaine et al., 2019). On the basis of these findings, this study proposes research hypothesis 3: creative self-efficacy mediates the relationship between challenging parenting behaviors and children’s creative tendencies.

### 1.4 Positive emotion and creative self-efficacy

Positive emotions may predict a sense of creative self-efficacy. According to the Self-efficacy Theory, self-efficacy plays a subjective role through cognitive processes, which are usually accompanied by motivational factors or processes (Gao, 1998). Bandura (1982) notes that emotional and physical conditions affect the development of self-efficacy. Existing research also suggests that positive emotions, such as pleasure and self-confidence, will enable individuals to maintain an optimistic and positive mindset in the face of difficulties, setbacks and failures, and to have the interest, confidence, courage, and motivation to think and act creatively, and thus be more likely to achieve positive outcomes, thereby creating a virtuous cycle with positive effects on self-efficacy (Puente-Diaz and Arroyo, 2016, 2018). In contrast, negative emotions, such as anxiety and worry, will cause individuals to underestimate their own ability level and find it challenging to perform the things and tasks they are engaged in, resulting in a heightened sense of incompetence, forming a vicious circle and negatively affecting self-efficacy (He and Wong, 2022). In addition, Woodman et al. (1993) argued that individual and situational factors interact to influence creativity. Consequently, children’s perceptions of their environment and others influence the development and maintenance of their creative self-efficacy. This means that when children perceive a positive external environment and positive feedback from others, they will be in a positive emotional state and are more likely to acquire a sense of creative self-efficacy, which provides motivation and incentive to think creatively and engage in creative activities that are challenging or innovative in nature (Yang and Zhang, 2012), thereby reinforcing their creative tendencies. In summary, this study proposes research hypothesis 4: challenging parenting behaviors are linked to children’s creative tendencies through the mediating roles of positive emotion and creative self-efficacy.

**FIGURE 1:**
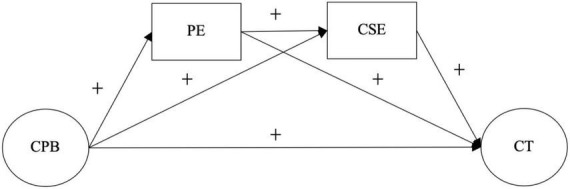
Conceptual framework of positive emotion and creative self-efficacy as mediators.

Based on the results of previous studies on challenging parenting behaviors and children’s creative tendencies, this study aims to investigate whether children’s positive emotions and creative self-efficacy mediate the relationship between challenging parenting behaviors and children’s creative tendencies. Based on the Social Cognitive Theory and the Self-efficacy Theory, an integrated hypothesis model (Figure 1) was proposed to reveal the complex associations. In this study, the following hypotheses were proposed: First, challenging parenting behaviors would positively influence children’s creative tendencies (H1). Second, children’s positive emotions will mediate the relationship between challenging parenting behaviors and children’s creative tendencies (H2), implying that challenging parenting behaviors may be a positive predictor of children’s positive emotions, which may in turn increase children’s creative tendencies. Then, children’s creative self-efficacy mediates the relationship between challenging parenting behaviors and children’s creative tendencies (H3), implying that challenging parenting behaviors may be a positive predictor of children’s creative self-efficacy, which may in turn increase children’s creative tendencies. Finally, challenging parenting behaviors influence children’s creative tendencies through a chain mediating effect of child positive emotions and child creative self-efficacy (H4), implying that challenging parenting behavior may be a positive predictor of child positive emotions, and child positive emotions may positively predict child creative self-efficacy, which may increase children’s creative tendencies.

## 2 Materials and methods

### 2.1 Participants

From September 1st to September 20th, 2022, we collected student and parent data through an online survey questionnaire in 13 primary and secondary schools in Shanxi Province, inland China. To ensure the validity of the questionnaire, we contacted the principals of the schools and explained the purpose of our research. We also provided advance explanations to parents and children about the content of the study-related questions and were available to answer any questions or concerns during their participation to ensure the accuracy of the research results. The distribution of the questionnaire was authorized by the principals, and informed consent was obtained from parents and students. To ensure that students and parents filled out the questionnaire voluntarily according to their true intentions, we first requested their voluntary participation in the research project. The participating students and parents were able to complete the survey in a calm and undisturbed environment. The questionnaire was administered anonymously to minimize external pressure on participants’ responses. The study randomly selected 13 primary and secondary schools and distributed 3,500 student questionnaires and 3,500 parent questionnaires. The response rate for student questionnaires was 98%, and the response rate for parent questionnaires was 97.79%. We excluded 587 invalid student questionnaires (obviously incorrect or not matched to the respondents) and 580 invalid parent questionnaires, resulting in 2,647 valid student questionnaires and 2,647 valid parent questionnaires. Table 1 shows demographic information, among the parents, there were 2,248 female respondents (mothers, 84.93%) and 399 male respondents (fathers, 15.07%). Among the students, there were 1,322 female respondents (girls, 49.94%) and 1,325 male respondents (boys, 50.06%). The response rate of the survey questionnaire was 75.63%. The average age of the minors was 12.58 years (SD = 1.67). All measures and procedures were authorized by the Institutional Review Board (IRB) of the first author’s institution.

**TABLE 1:** Demographic information.

Variables	Types	Number	Percent (%)
Gender of children	Male	1,325	50.06
	Female	1,322	49.94
Gender of parent	Male	399	15.07
	Female	2,248	84.93
Children’s educational level	Primary school	959	36.23
	Junior high school	1,688	63.77

### 2.2 Measures

#### 2.2.1 Challenging parenting behavior

In this study, challenging parenting behaviors were investigated using the English version of the Parent Challenging Parenting Behavior Questionnaire (CPBQ 4-6), revised by Majdandžić et al. (2018). This questionnaire consists of 39 items, including six dimensions: teasing, rough-and-tumble play, encouragement of risk-taking, social daring, competition, and modeling. It is scored on a five-point scale, with “not at all” scoring 1, “basically not” scoring 2, 3 points for “not sure,” 4 points for “mostly conform,” 5 points for “fully conform,” and reverse scores for questions 3 and 8. The higher the score, the higher the level of challenging parenting. This measure had a Cronbach’s alpha coefficient of 0.931 and Majdandžić et al. (2018) confirmed that the structural validity index was good [χ^2^/df = 1.53, comparative fit indices (CFI) = 0.973, root mean square error of approximation (RMSEA) = 0.059].

#### 2.2.2 Creative tendencies

In this study, children’s creative tendencies were measured using the Williams Creative Tendency Scale, revised by Lin and Wang (1999). The scale consists of 50 items, including four dimensions of risk-taking, curiosity, imagination, and challenge, and is scored on a 3-point scale, with “not at all” scoring 1, “partially” scoring 2, and “fully” scoring 3 points, and questions 4, 9, 12, 17, 29, 35, 45, and 48 were scored in reverse. Children with higher scores demonstrate more creative tendencies. This measure had a Cronbach’s alpha coefficient of 0.933 and the structural validity index was good [χ^2^/df = 9.827, RMSEA = 0.059, standard root mean square residuals (SRMR) = 0.068]. And Huang et al. (2021) measured the validity of the Williams Creative Tendency Scale using the Torrance Test of Creative Thinking as the criterion, confirmed that it has good reliability and validity.

#### 2.2.3 Positive emotion

In this study, positive affect in children was measured using the Positive/Negative Affect Scale (PANAS) devised by Watson et al. (1988), and the Chinese version of PANAS was revised and introduced by Huang et al. (2003). The scale consists of 20 adjective entries reflecting emotions, including two subscales of positive and negative emotions, and is scored on a five-point scale, with “almost none” scoring 1, “relatively little” scoring 2, “moderate” scoring 3, “relatively much” scoring 4, and “extremely much” scoring 5. The higher the positive emotion score, the more energetic the individual is and the happier and more focused the emotional state is; the higher the negative emotion score, the more subjectively confused the individual feels and the more distressed the emotional state is (Zhang, 2001). This measure had a Cronbach’s alpha coefficient of 0.817 and the structural validity index was good (χ^2^/df = 18.511, CFI = 0.94, RMSEA = 0.083).

#### 2.2.4 Creative self-efficacy

In this study, children’s creative self-efficacy was measured using the Creative Self-Efficacy Scale (CSE) of the Short Form of Creative Self (SSCS), which was devised by Watson et al. (1988). The scale has six items and is scored on a five-point scale, with “not at all” scoring 1, “not basically” scoring 2, “not sure” scoring 3, “basically” scoring 4, and “fully conforming” scoring 5. The higher the score, the higher the child’s sense of self-efficacy for creativity. This measure had a Cronbach’s alpha coefficient of 0.903 and the structural validity index was good (χ^2^/df = 8.413, CFI = 0.991, RMSEA = 0.054).

### 2.3 Statistics

Statistical analysis in this study was conducted using SPSS 22.0 and Mplus 8.3. Initially, Harman’s single-factor analysis was first performed to test for common method bias. Second, descriptive statistics and Pearson correlation analysis were performed with SPSS 22.0 to estimate the means, standardized deviations, and correlations among the main variables. Considering that child gender, age, and parental gender may have additional effects on parental challenging parenting behaviors, we decided to control for these variables in our analyses. Third, structural equation modeling (SEM) in Mplus 8.3 was used to examine the mediating effects of positive emotions and creative self-efficacy on challenging parenting behaviors and children’s creative tendencies. We calculated the following fit statistical scores to determine the degree of fit between the survey data and the hypothesized model: Chi-square (χ^2^) tests for differences, CFI, Tucker Lewis fit index (TLI) with values greater than 0.90, RMSEA with values less than 0.08, and SRMR with values near 0.05 are indicators of a good fit (Wen et al., 2004). The sample was also repeated 1,000 times using bootstrapping to test the mediating effects of positive emotions and creative self-efficacy on parental challenging behaviors and children’s creative tendencies.

## 3 Results

### 3.1 Common method variance analysis

Since the data relied on the subjective self-reports of parents and children, there may be covariates, so it is necessary to examine common method bias. In this study, the Harman’s single-factor analysis for common method bias (Harman, 1976) was utilized. The test revealed 18 factors with characteristic roots greater than one, and the variance explained by the first common factor was 18.47%, which is significantly less than the empirical criterion of 40% (Podsakoff et al., 2003). Therefore, there was no significant common method bias in this study.

### 3.2 Descriptive and correlation analyses

Table 2 provides the means, standard deviations, and correlation coefficients for each variable. Spearman’s correlations displayed that challenging parenting behaviors were positively and significantly correlated with children’s positive emotion (*r* = 0.30), children’s creative self-efficacy (*r* = 0.32), and children’s creative tendencies (*r* = 0.43), children’s positive emotions were positively and significantly correlated with children’s creative self-efficacy (*r* = 0.64), and children’s creative tendencies (*r* = 0.48), children’s creative self-efficacy were positively and significantly correlated with children’s creative tendencies (*r* = 0.56). In addition, there were significant positive correlations between children’s positive emotions, children’s creative self-efficacy, and each of the sub-dimensions of challenging parenting behaviors; between children’s positive emotions, children’s creative self-efficacy, and each of the sub-dimensions of children’s creative tendencies; and between each of the sub-dimensions of challenging parenting behaviors and each of the sub-dimensions of children’s creative tendencies.

**TABLE 2:** Descriptive statistics and correlation analysis of the main study variables.

	1	2	3	4	5	6	7	8	9	10	11	12	13	14	15
1. Children’s age	–														
2. Challenging parenting behavior	−0.14***	–													
3. Teasing	−0.09***	0.74***	–												
4. Rough-and-tumble play	−0.13***	0.80***	0.66***	–											
5. Encouragement of risk taking	−0.12***	0.80***	0.46***	0.55***	–										
6. Social daring	−0.14***	0.81***	0.45***	0.52***	0.69***	–									
7. Competition	−0.08***	0.81***	0.45***	0.49***	0.57***	0.64***	–								
8. Modeling	−0.08***	0.83***	0.49***	0.52***	0.57***	0.66***	0.75***	–							
9. Positive emotion	−0.11***	0.30***	0.16***	0.19***	0.29***	0.31***	0.25***	0.24***	–						
10. Creative self-efficacy	−0.04*	0.32***	0.16***	0.20***	0.29***	0.32***	0.28***	0.28***	0.64***	–					
11. Creative tendencies	−0.11***	0.43***	0.31***	0.30***	0.35***	0.36***	0.36***	0.38***	0.48***	0.56***	–				
12. Adventurousness	−0.09***	0.40***	0.30***	0.30***	0.30***	0.32***	0.33***	0.35***	0.40***	0.48***	0.90***	–			
13. Curiosity	−0.08***	0.39***	0.26***	0.27***	0.33***	0.34***	0.33***	0.34***	0.48***	0.57***	0.93***	0.77***	–		
14. Imagination	−0.11***	0.39***	0.27***	0.28***	0.32***	0.33***	0.33***	0.36***	0.46***	0.53***	0.93***	0.78***	0.82***	–	
15. Challenging	−0.11***	0.37***	0.29***	0.26***	0.31***	0.30***	0.31***	0.33***	0.40***	0.47***	0.88***	0.72***	0.76***	0.77***	–
*M*	12.58	3.47	3.06	3.33	3.92	3.69	3.62	3.21	3.64	3.72	2.22	2.13	2.30	2.21	2.25
SD	1.67	0.62	0.78	0.88	0.78	0.65	0.76	0.81	0.68	0.75	0.31	0.35	0.37	0.35	0.30

All tests are two-tailed. *p < 0.05,

***p < 0.001.

### 3.3 Chain mediation model analysis

**FIGURE 2:**
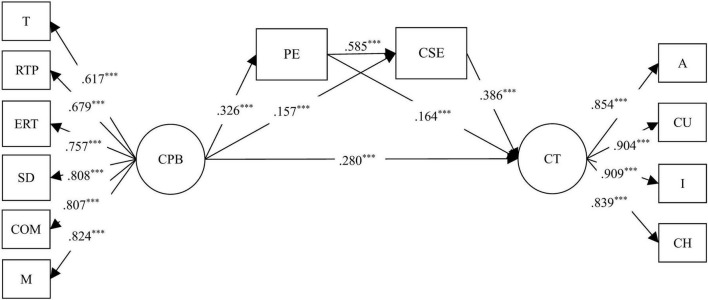
The chain mediation model. ***p < 0.001. CPB, challenging parenting behavior; CT, creative tendencies; PE, positive emotion; CSE, creative self-efficacy; T, teasing; RTP, rough-and-tumble play; ERT, encouragement of risk-taking; SD, social daring; COM, competition; M, modeling; A, Adventurousness; CU, Curiosity; I, Imagination; CH, Challenging.

Mplus 8.3 was used to examine the mediating effects of positive emotion and creative self-efficacy on the relationship between challenging parenting behaviors and children’s creative tendencies. Using the SEM method (Cheung, 2007), the chained mediation model demonstrated a satisfactory model fit (χ^2^/df = 23.42, *p* < 0.001, RMSEA = 0.08, CFI = 0.945, TLI = 0.928, SRMR = 0.036, *R*^2^ = 0.439). The non-parametric percentile bootstrap method with bias correction was utilized to evaluate the mediating effect. The bootstrap method with 1,000 bootstrap samples was utilized for the analysis. Figure 2 depicts a chain mediation model, and Tables 3, 4 present the SEM path coefficients and the mediating role of positive emotions and creative self-efficacy between challenging parenting behaviors and creative tendencies, respectively. The findings indicate that:

**TABLE 3:** Structural equation modeling path coefficients.

SEM path	Standardized		Non-standardized	
	**β**	**SE**	**β**	**SE**
Challenging parenting behavior to positive emotion	0.326***	0.022	0.457***	0.036
Challenging parenting behavior to creative self-efficacy	0.157***	0.02	0.243***	0.032
positive emotion to creative self-efficacy	0.585***	0.019	0.647***	0.023
Challenging parenting behavior to creative tendencies	0.280***	0.023	0.171***	0.015
positive emotion to creative tendencies	0.164***	0.024	0.071***	0.011
creative self-efficacy to creative tendencies	0.386***	0.028	0.152***	0.011

All SE are jackknifed standard errors. ***p < 0.001.

**TABLE 4:** Perceived organizational support and psychological empowerment in the mediating effect analysis.

Effect	Path relationship	Effect size	Bootstrap, 95% CI	Relative mediation effect (%)
Direct effect	Challenging parenting behavior → creative tendencies	0.280	[0.237, 0.323]	98.25
Path 1	Challenging parenting behavior → positive emotion → creative tendencies	0.033	[0.023, 0.044]	11.58
Path 2	Challenging parenting behavior → creative self-efficacy → creative tendencies	0.037	[0.026, 0.048]	12.98
Path 3	Challenging parenting behavior → positive emotion → creative self-efficacy → creative tendencies	0.045	[0.037, 0.056]	15.79
Total mediating effect		0.115	[−0.021, 0.015]	40.35
Total effect		0.285	[0.253, 0.321]	100
Compare 1		−0.004	[−0.021, 0.015]	
Compare 2		−0.012	[−0.028, 0.001]	
Compare 3		−0.008	[−0.02, 0.003]	

First, challenging parenting behaviors had a significant positive predictive effect on children’s positive emotions (β = 0.457, *t* = 12.779, *p* < 0.001), children’s positive emotions had a positive predictive effect on children’s creative tendencies (β = 0.071, *t* = 6.748, *p* < 0.001), challenging parenting behaviors positively predicted children’s creative tendencies (β = 0.171, *t* = 11.445, *p* < 0.001), and the mediating effect test revealed that the 95% CI [0.023, 0.044] did not include 0, indicating that children’s positive emotions mediated the relationship between challenging parenting behaviors and children’s creative tendencies, and the ratio of the mediating effect amount (0.033) to the total effect amount (0.285) was 40.35%, confirming hypothesis 2.

Second, the positive predictive effect of challenging parenting behaviors on children’s creative self-efficacy was significant (β = 0.243, *t* = 7.522, *p* < 0.001), and the positive predictive effect of children’s creative self-efficacy on children’s creative tendencies was significant (β = 0.152, *t* = 13.445, *p* < 0.001), and the mediating effect test revealed that the 95% CI [0.026, 0.048] did not include 0, indicating that children’s creative self-efficacy mediated the relationship between challenging parenting behaviors and children’s creative tendencies, with the ratio of the mediated effect size (0.037) to the total effect size (0.285) being 11.58%, confirming hypothesis 3.

Third, the positive effect of children’s positive emotions on children’s creative self-efficacy was statistically significant (β = 0.647, *t* = 28.612, *p* < 0.001), and the test of mediating effect revealed that the 95% CI [0.037, 0.056] did not include 0, indicating that children’s positive emotions and children’s creative self-efficacy play a chain role between challenging parenting behaviors and children’s creative tendencies. The ratio of the mediated effect size (0.045) to the total effect size (0.285) was 15.79%, confirming hypothesis 4.

## 4 Discussion

Our study contributes to the existing literature by investigating potential mechanisms between challenging parenting behaviors and children’s creative tendencies in an elementary and secondary school sample of Chinese children and their parents. This study examined a chain mediation model in which positive emotions mediated challenging parenting behaviors and creative tendencies, creative self-efficacy mediated challenging parenting behaviors and creative tendencies, and challenging parenting behaviors may directly influence children’s creative tendencies via the chain mediation effects of positive emotions and creative self-efficacy. Overall, these pathways provide a comprehensive and constructive perspective that elucidates the connection between challenging parenting behaviors and children’s creative tendencies. Additionally, the study provides preliminary evidence that the Social Cognitive Theory and the Self-efficacy Theory can be used to explain behaviors associated with children’s creative tendencies. The results will be discussed below.

### 4.1 The association between challenging parenting behavior and creative tendencies

The present study found that challenging parenting behaviors were directly and positively associated with children’s creative tendencies, which is consistent with the findings of Gralewski and Jankowska (2020) and Dong et al. (2022). Our hypothesis 1 holds. According to the Social Cognitive Theory, humans progressively acquire cognitive, emotional, and behavioral knowledge and skills through social interactions and the influence of their cultural environment. Thus, one possible reason for the direct and positive impact of challenging parenting behaviors on children’s creative tendencies is that early childhood is the peak of parental physical play with children (Pellegrini and Smith, 1998), and parents are able to stimulate and satisfy children’s curiosity and desire to explore external things and environments through daily challenging parenting behaviors, providing creative materials and challenging environments, thus prompting children to continuously learn new skills, acquire new experiences, and solve new problems. Simultaneously, children’s sense of competition, autonomy, exploration, self-confidence, and adaptability increase as a result of their parents’ daily challenging parenting behaviors (Paquette, 2004), and their cognitive, motor, social, and adaptive skills develop (Paquette et al., 2003; Bögels and Phares, 2008), all of which enhance children’s problem-solving skills and abilities, develop their creative thinking, and improve their creative abilities. The findings also provide additional evidence for the prominent role that challenging parenting behaviors play in the development of creative tendencies. Therefore, in the daily process of parenting, it is crucial for parents not to blindly shield their child from all harm or shield them from any frustration. Instead, they should engage in appropriate teasing and moderate play with their child, rather than treating them as a delicate “porcelain doll.” Additionally, parents can organize activities that require a certain level of effort to achieve specific goals, encouraging their children to think critically and develop the courage to explore new actions.

### 4.2 The mediating role of positive emotion

Consistent with previous research (Ren et al., 2017), the mediation analysis revealed that positive emotions mediated the association between challenging parenting behaviors and children’s creative tendencies. Children’s emotions are associated with parenting behaviors and parenting styles (Morris et al., 2010), and they are an important condition for the development of their creative tendencies. Positive emotions have a generally facilitating influence on children’s creative tendencies, whereas negative emotions such as anxiety and depression have an inhibiting effect (Ren et al., 2017). Specifically, positive emotions enhance children’s curiosity, imagination, and desire to investigate, increase their cognitive flexibility and autonomy, and thus increase the likelihood that children will think and act independently, fostering their creative tendencies. Therefore, children with positive emotions are more likely to participate in activities that require creativity. Parental support and encouragement embedded in challenging parenting behaviors can increase children’s self-confidence and performance abilities, reduce children’s anxiety (Möller et al., 2015; Lazarus et al., 2016; Smout et al., 2020), and have a positive effect on children’s mood (Möller et al., 2016). Positive emotions emerged as an important mediator in the study, further demonstrating the significance of positive emotions in fostering children’s creative tendencies. Research hypothesis 2 holds. Therefore, in the daily process of parenting, it is crucial for parents not to blindly shield their child from all harm or shield them from any frustration. Instead, they should engage in appropriate teasing and moderate play with their child, rather than treating them as a delicate “porcelain doll.” Additionally, parents can organize activities that require a certain level of effort to achieve specific goals, encouraging their children to think critically and develop the courage to explore new actions.

### 4.3 The mediating role of creative self-efficacy

Consistent with the Social Cognitive Theory, the findings identified creative self-efficacy as a significant mediator of the relationship between challenging parenting behaviors and children’s creative tendencies, which is consistent with prior research (Choi, 2004). The reason may be that in challenging parenting environments, parents provide children with moderate pressure and appropriate challenges to promote their growth and development (Grossmann et al., 2010), permit children to freely express their thoughts and feelings, fully respect children, and create a positive developmental environment for children. Parents also encourage children to push their limits through proactive physical and verbal behaviors (Majdandžić et al., 2016), and to do things they are usually afraid to do or fear to do, and to cope with fearful situations in a playful manner, so that children are braver when facing unfamiliar situations (Majdandžić et al., 2014), and to step outside of their comfort zone. According to the Self-efficacy Theory, when children receive positive support and feedback from their parents and are able to complete challenging tasks, they acquire confidence and a sense of identity, and their self-efficacy is enhanced (Cai, 2007). At the same time, the resulting strong belief and adventurous spirit will make children more likely to try to think and act in innovative and unconventional ways when confronted with new challenges and problems, which means that children gradually develop positive, confident, and independent creative thought, as well as the courage and freedom to explore and experiment on their own, thus enhancing their creative tendencies. Consequently, challenging parenting behaviors can indirectly affect children’s creative tendencies by influencing their creative self-efficacy. Research hypothesis 3 holds. The child’s growth process necessitates parents’ patience when the child encounters problems and attempts various problem-solving approaches. It is crucial for parents not to rush or substitute the child in solving the problem, but rather assist them when they seek help, while also providing encouragement and support to persevere.

### 4.4 The chain mediating effects of positive emotion and creative self-efficacy

Finally, the present study found that challenging parenting behaviors can increase children’s creative tendencies by fostering positive emotions and creative self-efficacy, and positive emotions and creative self-efficacy played a chain mediating role between challenging parenting behaviors and creative tendencies, which is consistent with our hypothesis 4 and validates the Social Cognitive Theory and the Self-efficacy Theory. According to research findings, challenging parenting behaviors, as well as positive and supportive parenting behaviors, can foster children’s creative tendencies. Specifically, when parents use positive parenting behaviors that are encouraging, supportive, and moderately challenging, the positive emotions that children experience as a result of their challenging parenting behaviors will motivate children to think or act spontaneously and creatively, continuously increasing children’s creative self-efficacy in the face of problems and challenges, which directly influences children’s responses and processing of new situations and problems, which is consistent with the Social Cognitive Theory. With parental encouragement, support, and assistance, children successfully adapt to and manage new situations and problems; their creative self-efficacy increases; their willingness and capacity to think and act in a creative manner increases; and their creativity improves, which is consistent with the Self-efficacy Theory. Therefore, in the daily process of parenting, it is crucial for parents to view their children as autonomous individuals. Particularly in Chinese families, parents should relinquish their “big parent” identity and grant children greater autonomy to choose and explore independently. Simultaneously, parents must serve as both an “umbrella” and a “leading light,” encouraging children to embrace challenges with courage while also reflecting deeply on problems from multiple perspectives. By stepping outside of their comfort zones and providing ample protection, encouragement, and love, parents can help their children grow into confident adults.

This study also found that children’s creative self-efficacy contributed more to their creative tendencies (β = 0.386, *p* < 0.01) than positive emotions (β = 0.164, *p* < 0.01). This may suggest that children’s creative tendencies derive primarily from their sense of creative self-efficacy during interactions with parents who adopt challenging parenting behaviors, as they perceive themselves to be capable of coping with difficult or innovative problems and challenges (Dollinger, 2011). In conclusion, this study contributes to the research on children’s creativity to some extent and provides empirical evidence that parents can provide children with positive emotional experiences through challenging parenting behaviors, thereby enhancing children’s creative self-efficacy and, consequently, their creative tendencies.

## 5 The theoretical and practical implications

In the traditional Chinese family parenting culture, Chinese parents may tend to choose a more strict parenting style that emphasizes discipline, rules, respect for elders, and authority (Mimi Chang, 2007). They also place a high emphasis on their children’s academic achievements and focus on cultivating their knowledge and skills. Therefore, this study may have certain implications and significance for Chinese parents. Specifically, the research’s implications are both theoretical and practical. Theoretically, this study contributes to the literature in two ways. On the one hand, this study suggest that challenging parenting behaviors have positive effects on children’s creative tendencies, which may contribute to a greater understanding by Chinese parents of the mechanisms underlying the relationship between parenting behaviors and children’s creativity. Specifically, it is possible for Chinese parents to increase their children’s willingness and propensity to think and act creatively by adopting challenging parenting behaviors that are motivating, supportive, and moderate (Lim and Smith, 2008). On the other hand, the study demonstrates that the mediating effects of positive emotions and creative self-efficacy can explain the relationship between challenging parenting behaviors and children’s creative tendencies, thereby enriching the literature on child creativity research. This means that positive emotions and creative self-efficacy can substantially transmit the beneficial effects of challenging parenting behaviors on children’s creative tendencies. Children require an environment that encourages and rewards creative thought in order to develop their creativity (Zhang and Sternberg, 2011). In challenging parenting environments, children could have a positive emotional state, a greater sense of creative self-efficacy, and stronger creative tendencies. Specifically, challenging behaviors by Chinese parents, such as teasing, rough-and-tumble play, and encouragement of risk-taking can foster positive emotions and creative self-efficacy in children, thereby reinforcing their creative tendencies. Actually, these findings may help Chinese parents and other child caregivers comprehend children’s creative tendencies from the perspective of environmental factors (e.g., challenging parenting behaviors) and children’s own characteristics (e.g., positive emotions and creative self-efficacy).

Regarding challenging parenting behaviors, Chinese parents should receive the necessary counseling, lectures and seminars, and appropriate support and guidance to help them recognize the importance of challenging parenting behaviors, increase their awareness and skills to engage in challenging parenting behaviors, and avoid overly easy or overly difficult challenges. In terms of positive emotions, Chinese parents should provide children with a challenging parenting environment in parent–child interactions, parental support such as encouragement, and recognition at the appropriate time, take care of children’s emotional state at all times, and adjust their own challenging parenting behaviors in time to help children develop their own creative and problem-solving abilities. For example, Chinese parents in their parent–child interactions can avoid treating their children as “porcelain dolls” to be carefully protected, but instead engage in playful activities with their children. They can also change the traditional Chinese parenting style of being reluctant to express love and appreciation, and instead frequently praise and encourage their children, openly complimenting them to enhance their self-confidence and resilience. Regarding creative self-efficacy, Chinese parents should provide children with challenging parenting behaviors that are appropriate to their developmental level and needs. By providing children with meaningful challenges and opportunities to acquire new skills, obtain new experiences, and solve new problems, children’s beliefs and expectations of self-creative performance are increased (Tierney and Farmer, 2002), thereby fostering their creative tendencies. For example, Chinese parents, when their children face everyday life problems or other issues, should avoid rushing to lecture and solve the problems for them. Instead, they should encourage their children to come up with multiple solutions and then allow the children to handle the situations themselves or provide some assistance as needed.

## 6 Limitations and future research directions

This research has several limitations. First, cross-sectional research cannot establish causal relationships across models; longitudinal and experimental investigations are required to confirm these associations. Second, this study regarded challenging parenting behaviors as a single variable and did not distinguish the influence of specific dimensions on children’s creative tendencies. Future research should investigate whether the internal mechanisms of the influence of particular dimensions on children’s creative tendencies vary. Finally, when only self-reported data are collected, sample bias may exist; the study’s validity can be evaluated further. Therefore, multiple measures, such as third-party observations, are required to reduce bias and improve reliability.

## 7 Conclusion

Children’s creative tendencies are one of the most significant manifestations of their imagination and creative abilities, as well as a crucial aspect of their fundamental literacy development. This study examines the potential mechanisms between challenging parenting behaviors and children’s creative tendencies. Challenging parenting behaviors are a set of measurable characteristics that children directly experience when interacting with parents who engage in challenging parenting behaviors and have an effect on children’s cognition, emotion, and behavior during parent–child interactions. Through a separate indirect pathway involving the stimulation of children’s positive emotions or creative self-efficacy, the study found that challenging parenting behaviors could positively predict children’s creative tendencies. Additionally, challenging parenting behaviors may also be associated with children’s creative tendencies via the chain-mediating effect of children’s positive emotions and creative self-efficacy. These findings provide a theoretical foundation for enhancing children’s creative tendencies and practical guidance for parents and other child caregivers to implement challenging parenting behaviors in order to foster children’s creative tendencies. In summary, this study contributes in the following ways: firstly, it investigates the relationship between challenging parenting behaviors and children’s creative inclination in the context of China based on social cognitive theory and self-efficacy theory, providing evidence for similar research in other countries. Secondly, by emphasizing the chain mediation effect of positive emotions and creative self-efficacy, it examines the mechanism of how challenging parenting behaviors influence creative inclination, offering a new perspective that explains children’s creative inclination being primarily influenced by positive emotions and creative self-efficacy (individual factors) originating from challenging parenting behaviors (environmental factors), thus providing effective recommendations for parents to enhance children’s willingness for innovation and development of creative abilities at the family level.

## Data availability statement

The raw data supporting the conclusions of this article will be made available by the authors, without undue reservation.

## Ethics statement

The studies that involve human participants have undergone review and approval by the Institutional Review Board (IRB) of Minzu University of China. Each participant in the study has provided informed, voluntary, and consensual consent to participate. The studies were conducted in accordance with the local legislation and institutional requirements. Written informed consent for participation in this study was provided by the participants’ legal guardians/next of kin.

## Author contributions

DS: Writing – original draft. YW: Writing – original draft. RJ: Writing – review & editing. LC: Writing – review & editing.
